# *Staphylococcus aureus* and MRSA Colonization Rates among Gravidas Admitted to Labor and Delivery: A Pilot Study

**DOI:** 10.1155/2007/70876

**Published:** 2007-12-26

**Authors:** R. Beigi, J. Hanrahan

**Affiliations:** ^1^Department of Obstetrics, Gynecology & Reproductive Sciences, Magee-Womens Hospital, University of Pittsburgh School of Medicine Pittsburgh, PA 15213, USA; ^2^Department of Medicine, Division of Infectious Diseases, MetroHealth Medical Center, Case Western Reserve University School of Medicine, Cleveland, OH 44109, USA

## Abstract

*Objective*. To determine colonization rates of *Staphylococcus aureus* given the potential for future intervention trials aimed at reducing surgical-site infectious morbidity, and to estimate methicillin-resistant *Staphylococcus aureus* (MRSA) rates in our patient population. *Study design*. Prospective pilot investigation comprising data from 104 gravidas admitted to an urban labor and delivery unit. All underwent anterior nares culture collection with a subset also undergoing vaginal culture collection. *Results*. Twenty-two percent of women were colonized in the anterior nares. Of the 28 women who had vaginal cultures collected, 4/28 (14.2%) demonstrated *Staphylococcus aureus* colonization. There was 82% concordance between the nares and vagina. Nine percent of isolates were MRSA strains. Overall, 2/96 (2.1%) of women were MRSA-colonized. *Conclusions*. Rates of *Staphylococcus aureus* colonization among gravidas entering labor and delivery are modest and consistent with the general population. MRSA rates among gravidas appear to be reassuringly low in this pilot study.

## 1. INTRODUCTION

*Staphylococcus aureus* is a common bacterial pathogen frequently found to colonize skin, anterior nares, or perineum in humans. Nasal carriage rates are 25–50% in the general population [1, 2]. *S. aureus* is a major cause of skin and surgical-site infections, and is one of the most common causes of healthcare-associated infections. Methicillin-resistant *S. aureus* (MRSA) has been a growing problem in healthcare facilities since the 1960s, and has become gradually more difficult to treat due to increasing resistance [3].

MRSA was once considered to be a problem primarily related to transmission in healthcare facilities due to cross-transmission on the hands of healthcare workers [4]. However, in the last ten years, there have been multiple outbreaks MRSA in people with no direct contact with healthcare settings, and no apparent risk factors for acquiring MRSA [5]. Community-acquired MRSA (CA-MRSA) infections have been increasingly reported, including invasive infections in children, outbreaks in correctional settings, athletic teams, and among men who have sex with men [5]. CA-MRSA infection has been recognized worldwide, and its increase poses serious implications for hospitals in that the pool of individuals colonized with MRSA will likely increase with subsequent increased potential for spread in hospitals.

The carriage rate for *S. aureus* and MRSA among women presenting for obstetric care has not been recently evaluated. An evaluation published in 1978 of *S. aureus* nasal colonization among asymptomatic gravidas admitted to labor and delivery documented a 4% colonization rate [6]. The importance of *S. aureus* colonization as a marker for subsequent surgical-site infectious morbidity is well documented [7, 8]. *S. aureus* is thought to be a causative agent in roughly 25–50% of cesarean section infectious wound morbidity and puerperal mastitis [9]. With the ever-increasing rates of cesarean delivery, recognition of potentially modifiable risk factors for surgical-site infectious morbidity becomes imperative.

MRSA is a cause of invasive disease in infants in neonatal intensive care units. The existence of MRSA colonization in pregnant women has potential serious implications for newborns [10–12], and may cause an increased rate of infection in both pregnant women and infants. Despite the potential implications, a paucity of data currently exists addressing MRSA rates among gravidas. In light of these concerns a pilot investigation into the prevalence of *S. aureus* and MRSA colonization in women presenting to the labor and delivery for labor management or scheduled cesarean section was undertaken.

## 2. METHODS

### 2.1. Patient population

This was a prospectively enrolled cohort analysis of 104 gravidas admitted to labor and delivery for labor management or scheduled cesarean section from April 2005 thru March 2006 at MetroHealth Medical Center (Cleveland, Ohio, USA). MetroHealth Medical Center is the Cuyahoga County public hospital, serving predominantly the inner-city metropolitan Cleveland area. The protocol was approved by the MetroHealth Medical Center institutional review board and all patients underwent informed consent.

Women who met the following inclusion criteria were approached for enrollment by a trained member of the research staff: gestational age at or beyond 24 weeks, were being admitted for labor management or scheduled cesarean section, and had intact amniotic membranes. Women were excluded if they had used antibiotics in the week preceding enrollment or had already received a pelvic exam that day with the use of bacteriostatic lubricant gel. At enrollment, demographic data including age, race, gestational age, and occupation including contact with health-care facilities or health-care personnel was obtained. All women had anterior nares swabs collected for *S. aureus* culture, and a subset also had swabs collected from the outer third of the vagina for *S. aureus* culture. Women received their regular care as per obstetric indication and no further follow-up took place during the incident hospitalization.

At 3 months postpartum the comprehensive clinical care computer database was searched for any visits the enrolled women received in the MetroHealth system after delivery pertaining to infectious morbidity. In addition, all women were contacted by phone by the trained research assistant to inquire into infectious conditions they experienced since delivery that may have been attributable to *S. aureus*. Specifically, women were asked if they had been diagnosed by a health professional with either a surgical wound infection (for cesarean delivery patients) or mastitis. Women who reported puerperal infectious morbidity to the research personnel on the phone or who were noted to have had a visit in the computerized medical record addressing one of these infections were compared to women without, stratified by *S. aureus* colonization status.

### 2.2. Microbiology

Swabs from both the anterior nares and vagina were cultivated for *Staphylococcus aureus* in the following manner at the MetroHealth Medical Center microbiology laboratory: swabs were plated on TSA II 5% Sheep's Blood agar (manufacturer: BBL). Plates were then incubated at 37°C in 5% CO2 and examined daily for growth. Colonies showing a typical *Staphylococcus* morphology were tested for bound coagulase using a slide agglutination test with rabbit plasma “clumping factor.” Colonies demonstrating positive agglutination in plasma were retested with a saline-negative control to confirm absence of autoagglutination **.** Equivocal “clumping factor” tests were resolved with a Tube Coagulase Test read at 24 hours of incubation. The plates were held 72 hours before cultures were resulted as negative. *S. aureus* isolates, then, underwent antimicrobial susceptibility testing via broth microdilution using a Vitek I. MRSA strains were identified as those demonstrating an MIC value of ≥ 4mg/dL to oxacillin (Clinical and Laboratory Standards Institute (CLSI), Performance standards for antimicrobial susceptibility testing (Wayne, Pa, USA); 2005).

Collation and analysis of the data was performed using StatView (version 5.0.1, SAS Institute Inc., (Cary, NC, USA)). Summary statistics were used for description of the data where appropriate. Fishers’ exact testing for differences in proportions was used where appropriate.

## 3. RESULTS

Of the 104 women enrolled, culture data is available on 96 (92.3%).

This represents approximately 3.5% of the women delivering at the institution over the time period of the study. The demographic data is displayed in Table 1. Most of the women presented at or near term, and a large percentage were admitted for scheduled repeat cesarean section delivery.

Of the 96 women with culture data, 21 (22.0%) were colonized with *S. aureus*. All but one (95.4%) of the culture-positive women carried *S. aureus* in the anterior nares. One woman harbored *S. aureus* in the vagina with a negative nares culture. There were seven of the nares-positive women that also had vaginal cultures taken, and 3 of 7 (42.8%) were correspondingly positive as well. Of the 75 women that were culture-negative in the nares, 21 of them also had vaginal cultures available, and all but one were culture-negative. Overall, of the 28 women with both nares and vaginal cultures available, 23 (82.1%) were concordant.

Two of the 21 (9.5%) women positive for *S. aureus* carried MRSA strains (2.1% overall). One of these women with both nares and vaginal cultures collected demonstrated MRSA colonization at both sites. Of the two MRSA (+) women, 1 worked in the study hospital and one denied any direct hospital/personnel contact other than outpatient prenatal care during the entire pregnancy prior to admission to labor and delivery.

In terms of infectious outcomes, 6 of the 96 women (6.3%) were noted to have infectious conditions potentially attributed to *S. aureus* (4 surgical wound infections and 2 cases of puerperal mastitis). Two of the 21 *S. aureus* (9.5%) colonized women had puerperal infectious morbidity versus 4 of 75 (5.3%), however, this did not reach statistical significance (*P* = .61).

## 4. DISCUSSION

This pilot investigation is the first study identified in nearly 30 years addressing *S. aureus* nasal colonization rates among otherwise uncomplicated gravidas entering labor and delivery. We documented a modest overall *S. aureus* colonization rate of 22%, of which nearly 10% were MRSA strains. The *S. aureus* nares colonization rate in this report approaches other published reports of the general population of 25–50% [1, 2]. These findings could serve as a foundation for future intervention trials using topical antimicrobials to decrease surgical-site infectious morbidity in patients increasingly undergoing scheduled cesarean delivery. Our overall MRSA colonization rate of 2.1% approximates the 0.8% rate noted from 2001–2002 national data [5]. This was a small pilot study and is informative although it may not be generalizable to the entire pregnant population.

The overall *S. aureus* nares colonization rate of 22.0% noted in this study is higher than the only other investigation addressing nasal colonization among asymptomatic gravidas, noting a 4.0% *S. aureus* nasal colonization in 1978 [6]. Chen et al. recently published *S. aureus* colonization rates from rectovaginal specimens collected for routine group B streptococcus (GBS) cultures done between 35–37 weeks of gestation and found that 17.1% of nearly 3000 women also had evidence for genital *S. aureus* colonization [13]. The subset of our women who had vaginal cultures performed (N = 28) showed a comparable rate of 4/28 (14.2%) genital tract S. aureus colonization. The main goal of this investigation was to delineate nasal *S. aureus* and MRSA colonization rates as a foundation for potential intervention trials using intranasal antimicrobials given the ever-increasing rates of cesarean delivery with its attendant surgical site morbidity. To this end, the vaginal colonization data was secondary, and is mentioned as a corollary to nasal colonization in a subset of women to address concordance in colonization sites.

Too few infectious outcomes with no direct incident culture data were detected in this pilot investigation to make any meaningful statements, however, it is worth noting the nearly 2 fold risk seen among women who were *S. aureus* culture-positive. Other patient populations have demonstrated an increase in surgical-site and soft-tissue infectious morbidity among those colonized with *S. aureus* [7, 8], but this has not been demonstrated to date in women undergoing cesarean section and/or lactating.

A related concern is the emergence and persistence of CA-MRSA strains in the general population. First recognized in the 1960s, MRSA has become an important pathogen not only due to its antibiotic susceptibility pattern making efficacious treatment challenging but also because of the severity of MRSA skin, soft-tissue, and blood-borne infections. Recent data suggests that CA-MRSA strains persist, placing women entering labor and delivery at risk for colonization, infection, and nosocomial transmission and/or acquisition of MRSA [5]. The rate of 2.1% in this pilot investigation approximates other reports and is reassuringly low, yet present nonetheless [5, 13].

A few limitations to the current pilot study are worth noting. This is a small study in a single locale, and thus may not be completely generalizable to the entire obstetric population. In addition, our method of assessing postpartum infectious morbidity was limited to patient report and/or search of an electronic medical record for visits. Thus, our estimates may not be entirely representative of reality. However, this was not done with knowledge of colonization category by the research personnel conducting the medical record search or the phone call, and is thus unlikely to be biased with regard to colonization status. Further, we did not investigate in this pilot study into the molecular characterization of our strains and therefore the epidemiology can only be suggested. However, of our 2 MRSA strain-positive women, 1 denied direct hospital contact except for outpatient prenatal care, making CA-MRSA a possibility.

In summary, this pilot study demonstrates modest and population-consistent rates of *S. aureus* and MRSA colonization rates among uncomplicated gravidas entering labor and delivery for management of labor. Given the increasing rates of cesarean delivery documented nationally, this population could potentially benefit from interventions aimed at reducing surgical-site infectious morbidity attributable to *S. aureus*. Continued surveillance for CA-MRSA is warranted among this and other “low-risk” populations due to increasing reports of prevalent strains in the community.

## Figures and Tables

**Table 1:** 

Demographics N-96		
Age (years)	

Mean	26	
Range	18–41	

Race n (%)	

Caucasian	46 (48%)	
A. A.	36 (38%)	
Latina	13 (13%)	
Asian	1 (1%)	

Gestational age (weeks)	

Mean	37	
Range	30–41	

Health-care exposure during pregnancy?	**N**	% Culture (+)

Yes	16 (17%)	25.0
No	80 (83%)	21.2

Delivery mode	**N**	% Culture (+)

Vaginal	33 (34%)	18.1
Cesarean	63 (66%)	23.8
